# The importance of PROSPERO to the National Institute for Health Research

**DOI:** 10.1186/2046-4053-1-5

**Published:** 2012-02-09

**Authors:** Sally Davies

**Affiliations:** 1 Chief Medical Officer and Chief Scientific Adviser, Department of Health, London, SW1A 2NS, UK

## Abstract

An important activity of the National Institute for Health Research is to commission and support the preparation of systematic reviews. For research commissioners, the international prospective register for systematic reviews known as PROSPERO provides an important further step in ensuring the quality and integrity of research evidence and will help avoid unplanned research duplication.

## Background

A key role of the National Institute for Health Research (NIHR) is to develop the research evidence to support decision-making by professionals, policy-makers and patients, to make this evidence available, and to encourage its uptake and use.

Decision-makers in health care, both clinicians (making decisions directly with their patients) and policy-makers, rely particularly on systematic reviews. These represent the highest level in a hierarchy of research evidence, especially when they combine the findings from multiple randomized controlled trials using meta-analysis. At a time when the amount of new published research evidence is increasing so rapidly, systematic reviews also provide an important practical way for professionals to remain up to date with developments in evidence in their fields.

## Discussion

The NIHR is making sure that the UK health service has access to the best possible evidence to inform decisions and choices by commissioning systematic reviews, by building capacity for their conduct and supporting the development of methods. Over the last 5 years, the NIHR has invested over £35 million in the infrastructure and related programs that are required within the UK to support the production of high-quality systematic reviews, and it will continue to invest in this key area.

However, resources are finite and, in the same way that health services must continually strive for effectiveness and efficiency in the treatments and services they provide, so research commissioners must ensure that waste is avoided in the development of research evidence. Investment in new research studies can only be justified when the questions they are addressing cannot be answered by existing evidence [1]. Systematic reviews provide an important way of defining unanswered questions in health and social care, and research commissioners are increasingly expecting applications for research funds to be supported by the findings of systematic reviews.

The NIHR has supported the development of International Register for Health Research, PROSPERO, by the NIHR's Centre for Reviews and Dissemination at the University of York. PROSPERO provides the first global online facility to register systematic reviews for research into health and social care, designed through widespread consultation, to be used internationally to promote best practice around the world.

For the National Health Service and the NIHR, the development of PROSPERO represents an important further step in ensuring the quality and integrity of research evidence. The transparency achieved through prospective registration of systematic reviews should promote high methodological standards and may help reduce selective reporting of research.

The register will also be an important tool for researchers and research commissioners who need to avoid unplanned and potentially wasteful duplication of systematic reviews. For those relying on systematic reviews to develop national guidance, such as the National Institute for Health and Clinical Excellence the register will provide important indications of what reviews are being carried out and when the findings are likely to become available.

## Conclusion

The NIHR encourages all those undertaking systematic reviews to register them prospectively with PROSPERO, and recommends the register to decision-makers and research commissioners as an invaluable source of information about what is happening in this key area of research endeavor.

## Competing interests

The author is the lead for Research & Development at the Department of Health, England and for the NIHR.

## References

[B1] Chalmers I Glasziou P Avoidable waste in the production and reporting of research evidence Lancet 2009 374 86 89 19525005 10.1016/S0140-6736(09)60329-9

